# Medical students’ perception on fecal microbiota transplantation

**DOI:** 10.1186/s12909-019-1804-7

**Published:** 2019-10-11

**Authors:** Petru C. Madar, Oana Petre, Adriana Baban, Dan L. Dumitrascu

**Affiliations:** 1 2nd Department of Internal Medicine, Iuliu Hatieganu University of Medicine and Pharmacy, Cluj-Napoca, Romania; 2 Department Psychology, Babes-Bolyai University, Cluj-Napoca, Romania

**Keywords:** Fecal transplantation, Medical education, Medical students

## Abstract

**Background:**

Fecal microbiota transplantation (FMT) has become an emergent method in the therapy of several intestinal diseases, mainly in *Clostridium difficile* recurrence. The training of FMT in medical schools is at its beginning and in countries where FMT is only occasionally carried out, it is important to know the perception of medical students on FMT.

**Methods:**

We undertook a survey of 3rd year medical students not exposed to official academic information on FMT in order to find out their knowledge, beliefs and attitude toward FMT. A number of 80 students were asked to fill a dedicated online questionnaire.

**Results:**

52 out of 80 third year medical students anonymously filled the questionnaire (65% response rate). 34% of respondents reported to have at least a medium level of knowledge regarding FMT. The top indication for FMT identified by 76.9% was *C. difficile* infection; however, 60% believed FMT to be a promising therapy for a high number of conditions and while almost all respondents (98.1%) would recommend it, 88.4% would explore other options first. Colonoscopy was considered the optimal method of delivery by 42.3%. Only 39% of participants believed that patients would accept FMT, however 71% considered that a more socially acceptable name for the procedure and anonymous donors would increase acceptance rate. The risk of transmitting a disease undetected by donor stool screening procedures to the recipient was the most worrying side effect considered by 75% of respondents. 54% believed that more research is required for FMT to enter clinical practice and 55.7% of respondents would enroll patients in controlled clinical trials.

**Conclusions:**

Medical students not exposed to educational information on FMT seem to be somewhat well informed about this method and would recommend it to their patients. Students, however, need to know more on the indications of FMT.

## Background

The vast diversity of the human gut microbiota includes more than 1000 species of bacteria and about 150 times more genes than the human genome [1]. Bacteria can be found throughout the entire length of the gastrointestinal tract, although with significant variability in composition and diversity. Most studies, however, focus on the microbiota obtained from stool samples [2]. The host-microbiota interaction has been extensively studied and several important homeostatic functions have been outlined. Thus, the microbiota has been found to be involved in metabolic processes (salvaging calories, production of short-chain fatty acids and certain amino-acids, vitamin K and folic acid synthesis, activation of drugs such as sulfasalazine), in bile acid deconjugation and in preventing the mucosa from being colonized by pathogens. The immunologic effects lead to increased production of immunoglobulin A and anti-inflammatory cytokines, as well as a down-regulation of proinflammatory cytokines and to regulatory T cells induction [3].

Alteration in the composition of the gut microbiota is known as dysbiosis and can be associated with various diseases. Although dysbiosis has been predominantly studied as being involved in the pathogenesis of gastrointestinal diseases, such as *C. difficile* infection (CDI) [4] and inflammatory bowel disease (IBD) [5], and in metabolic diseases [6], a growing body of evidence suggests involvement of microbiota in diseases of other systems [7].

Fecal microbiota transplantation (FMT) aims to restore eubiosis by transferring previously screened stool from healthy donors (either from an unrelated or related local donor or from a stool bank) to the gastrointestinal tract of affected individuals [8]. While not a novel medical procedure [9, 10], FMT is receiving increasingly more attention in recent years [11] and the strongest evidence of its efficacy comes from its application in treating recurrent *C. difficile* infection (rCDI). A recent systematic review of the literature that included 516 patients in 21 case-series and two randomized, controlled trials (RCT) observed a symptom resolution in 85% of cases [4].

Current donor screening procedures do not follow an established universal protocol; however, several protocols are commonly used [12]. Stool and serum are screened against infectious agents, including antibiotic-resistant bacteria. Age over 50, infectious diseases, personal or first-degree relative history of IBD or gastrointestinal malignancy, diabetes, obesity, recent travel to regions endemic for gastrointestinal pathogens and antibiotic use in the last 6 months represent commonly used donor exclusion criteria [13, 14].

Methods of performing FMT consist mainly of frozen oral capsules, enemas, nasojejunal tube delivery and direct delivery through a colonoscope, each presenting with its own advantages and side effects profile. From an efficacy standpoint, Kao et al. compared oral capsule- vs colonoscopy-delivered FMT in rCDI and found it to be non-inferior [15]. The use of frozen vs fresh FMT was also found to be non-inferior [16].

Several guidelines already recommend FMT in rCDI, however with differences regarding the strength of evidence. FMT is strongly recommended to be used starting with the second relapse by European guidelines [17], while American guidelines only recommend its use starting with the third relapse after a pulsed vancomycin regimen [18].

Currently, no Romanian guidelines on FMT exist as it is not routinely performed. The limited national experience with regard to performing FMT comes from two small studies. In one study, Laszlo et al. performed FMT on five selected cases of ulcerative colitis, with severity ranging from left colitis to pancolitis. Four patients had either failed or refused anti-TNFalpha therapy and one patient previously treated with azathioprine had tested positive for *C. difficile* toxins A and B. A fecal suspension was prepared from screened stool samples obtained from healthy relatives and was administered through a colonoscope. Three patients maintained clinical and biological remission at a one-year follow-up and one patient relapsed after 10 months [19].

In a similar study published by Oprita et al., 33 patients (28 with refractory *C. difficile* infection and 5 with severe ulcerative colitis unresponsive to anti-TNFalpha therapy) underwent FMT, either via colonoscopy or nasojejunal tube. Resolution of symptoms was achieved in all of the *C. difficile* patients and after 3 months of follow-up, only one patient relapsed, but was successfully treated with oral vancomycin. Symptom resolution was also achieved in 4 patients with ulcerative colitis, however 3 patients required repeat infusion. At a three-month follow-up, one patient died (no correlation to FMT) and one relapsed and required surgery [20].

Studies have shown that patient acceptance of FMT is high [21] and that they are not deterred by the potentially unappealing nature of the procedure [22]. Physicians are often reluctant to recommend the procedure, citing lack of evidence about efficacy [23], although the physicians’ acceptance rate appears to be high in China [24].

Future generations of physicians will need to have a clear view on FMT and how it could benefit their patients, however the perception of FMT among medical students has not, to our knowledge, been studied so far.

The aim of this study was to investigate the knowledge and attitudes of Romanian medical students about the FMT, in order to get information that can be used to further improve medical education.

## Methods

### Survey development

An online cross-sectional survey was carried out, using a convenience sampling method to obtain an overview of the medical students’ knowledge and attitudes towards FMT.

The survey instrument was developed following a literature review and discussions with gastroenterologists from Iuliu Hațieganu University of Medicine and Pharmacy Cluj-Napoca, Romania. The ad-hoc questionnaire was constructed to be completed in approximately 5 min and contained two main sections. Firstly, seven multiple-choice items were used to assess: knowledge about FMT (self-assessed level of expertise and specific knowledge about implications, methods and side effects), readiness to recommend the procedure and sources of information about FMT. The second part of the questionnaire evaluates attitudes toward FMT procedure. The attitudes scale consisted in 30 self-rated items, measured on a 5-point Likert scale ranging from 1 (strongly disagree) to five (strongly agree).

The questionnaire was built and hosted on Google Forms and a link was made available to students during their semiology rotation in our clinic. It was open between 01.05.2018 and 30.06.2018. No reward was offered for participating in this study. A PDF version of the questionnaire is provided as (Additional file 1).

### Study participants

The study participants were third year medical students from a single medical faculty that had no prior exposure to lectures on FMT. Participation was voluntary after informed verbal consent and anonymous. Eighty students were invited to fill the questionnaire. Fifty-two agreed to fill the questionnaire, giving a response rate of 65%.

### Analysis

We performed descriptive statistical analysis on all the submitted answers. Results are presented as frequency counts and percentages by category. Data analysis was performed using Microsoft Excel (Microsoft Excel 2016, Version 16.0, Microsoft Corporation, Redmond, WA).

## Results

### Self-reported degree of knowledge regarding FMT

A total of 52 students completed the online form. The median age was 21 years. Detailed results are provided as (Additional file 2). Regarding FMT knowledge, 34 and 30% of responders reported a very low and low degree, respectively. Another 30% reported to have a medium degree of knowledge. High and very high degrees of knowledge were each reported by 2% of participants.

### Sources of information

The top three most used sources of information concerning FMT were courses (73%), the internet (69%) and documentaries (66%). When asked about how frequently they use these sources, 21 to 31% of participants indicated that they rarely do so.

### FMT indications

**Fig. 1:**
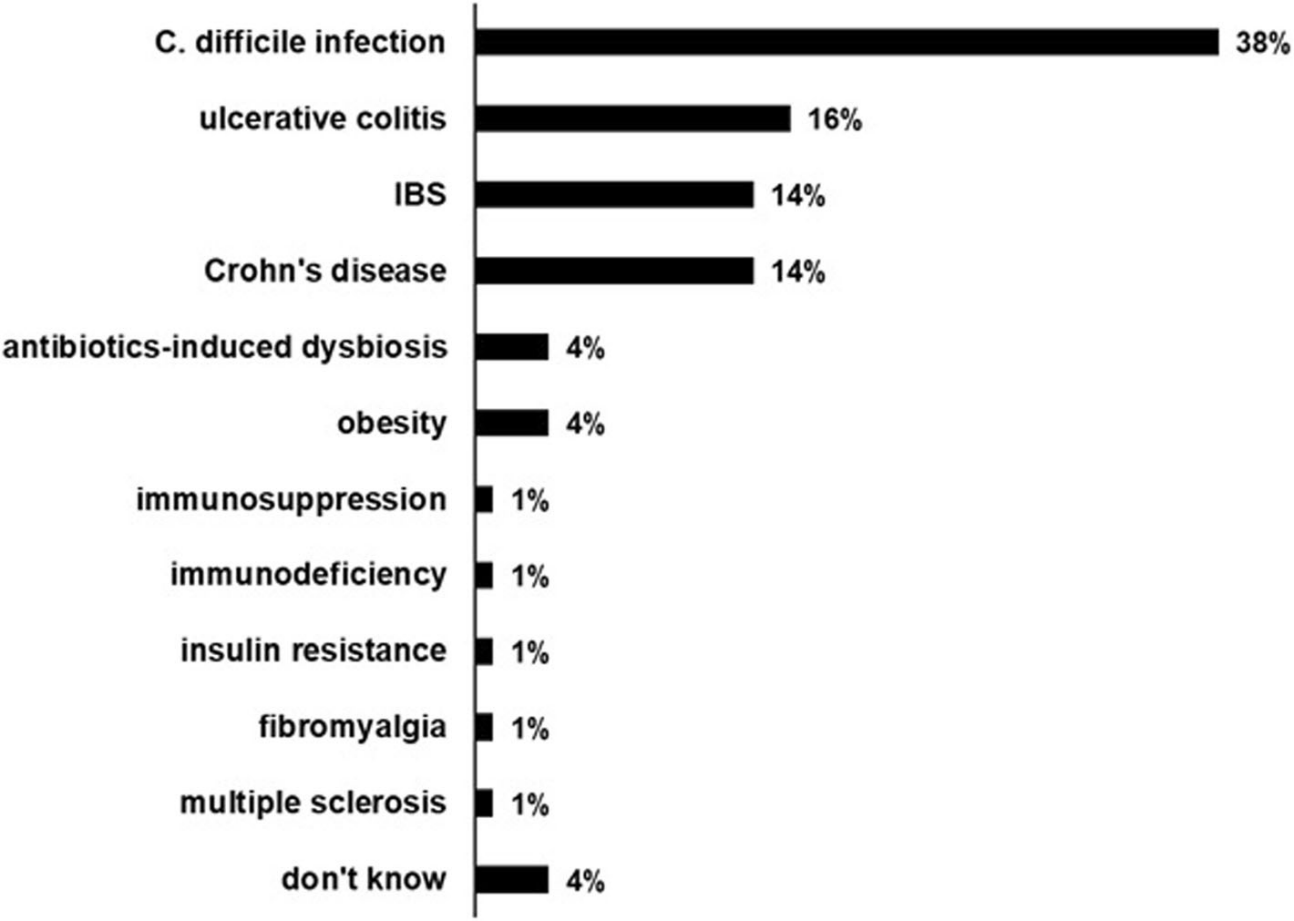
Fecal microbiota transplantation indications

The top indication for performing FMT identified by respondents was *Clostridium difficile* infection (76.9%), followed by ulcerative colitis (32.7%), Crohn’s disease (28.8%) and IBS (28.8%). Indications are summarized in Fig. 1.

### Side effects

The transmission of diseases previously undetected by screening procedures was the most worrying adverse effect considered by 75% of participants. Gastrointestinal symptoms such as bloating (57.7%), diarrhea (53.8%), abdominal discomfort (51.9%), constipation (36.5%), cramping (34.6%) and nausea (32.7%) were also considered.

### Optimal methods of performing FMT

Participants were asked about which method they consider to be the optimal way of performing FMT. Thus, 42.3% of respondents would use colonoscopy. Other considered procedures to deliver FMT were enemas (15.4%), nasojejunal tube (7.7%) and frozen oral capsules (5.8%).

### Readiness to recommend

**Fig. 2:**
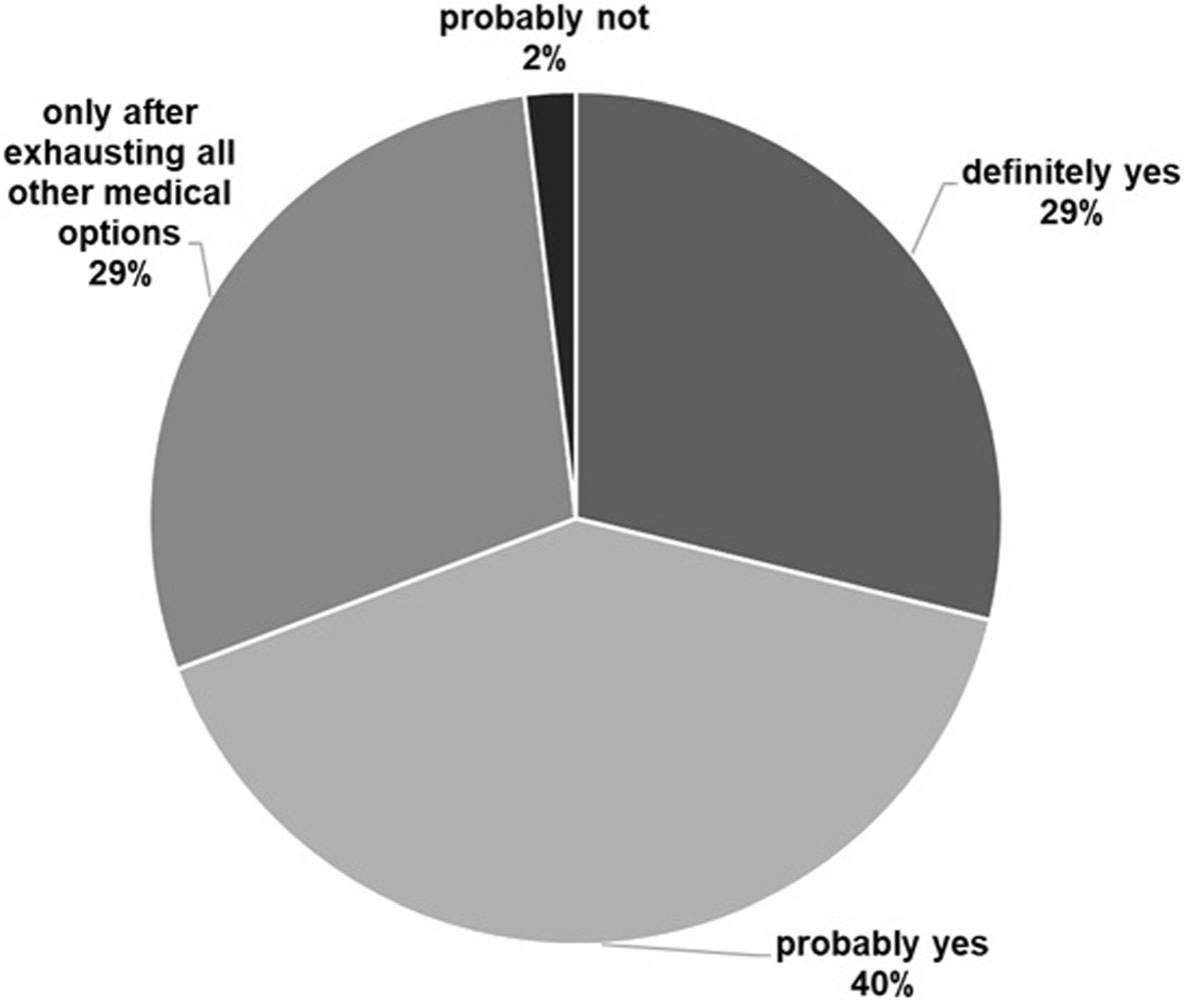
Willingness to recommend FMT

When asked whether they would recommend FMT to patients where such an indication exists, 98.1% of participants were likely to do so. Regarding timing, 34.6% of participants stated that other medical alternatives should first be explored before performing FMT vs. 9.6% who would readily opt for FMT as first line treatment. Willingness to enroll patients in controlled clinical trials was expressed by 55.7% of responders, 36.5% were not sure and 7.6% would be unwilling to do so (Fig. 2).

### Attitudes towards FMT

The distributions of responses to the 30 self-rated items are summarized in Table 1.

**Table 1:** Distributions of responses to 30 self-rated items concerning beliefs and attitudes towards FMT

	Self-rated item	Strongly disagree	Somewhat disagree	Neither agree, nor disagree	Somewhat agree	Strongly agree
1	I feel uncomfortable talking to patients about FMT	13%	37%	33%	15%	2%
2	A more socially acceptable name would make discussions about FMT easier	2%	4%	25%	48%	21%
3	I believe patients would accept FMT as treatment	10%	33%	42%	12%	4%
4	More controlled clinical studies on FMT are needed before it can be used in clinical practice	4%	23%	46%	19%	8%
5	I believe there is currently enough evidence for FMT	23%	37%	25%	10%	6%
6	I do not believe in the long-term results of FMT	19%	17%	21%	31%	12%
7	FMT is a risky procedure	4%	25%	56%	13%	2%
8	FMT is a long and complicated process	27%	38%	25%	8%	2%
9	FMT can lead to transmitting of infections	13%	21%	25%	29%	12%
10	FMT poses a high risk of disease exacerbation	12%	12%	48%	12%	17%
11	FMT does not represent a permanent solution to the medical problem	2%	21%	46%	19%	12%
12	FMT belongs to alternative medicine	29%	29%	40%	2%	0%
13	I find FMT somewhat disgusting	2%	15%	38%	35%	10%
14	FMT is a safer treatment that standard medical treatments	2%	10%	29%	48%	12%
15	FMT is a riskier medical procedure than blood transfusion	15%	12%	38%	21%	13%
16	FMT has a negative impact on patient dignity (feelings of shame, stigma, etc.)	4%	23%	42%	19%	12%
17	I believe the procedure would be supported within my institution	21%	33%	35%	10%	2%
18	Results from veterinary medicine represent strong evidence for using FMT in human medicine	6%	8%	25%	25%	37%
19	FMT can do more harm than good	2%	17%	35%	23%	23%
20	FMT is a therapy comparable to probiotics	10%	19%	37%	27%	8%
21	I believe FMT is a promising therapy for many diseases	2%	12%	37%	31%	19%
22	I believe this procedure would be covered by insurance in the future	4%	15%	27%	37%	17%
23	Donor screening is a complex and costly procedure	0%	12%	17%	31%	40%
24	It’s easier for someone to be “admitted to Harvard” than fulfill the criteria to become a stool donor	13%	37%	33%	15%	2%
25	I am interested in learning more about FMT and its practice	2%	4%	25%	48%	21%
26	I believe the procedure should only be carried out in medical centers specialized in FMT	10%	33%	42%	12%	4%
27	I believe FMT should become a usual procedure in every clinic/hospital	4%	23%	46%	19%	8%
28	I believe the procedure would be more accessible (duration, costs, logistics) if a stool bank existed	23%	37%	25%	10%	6%
29	I believe the procedure would be more easily accepted by doctors and patients if a stool bank existed	19%	17%	21%	31%	12%
30	I believe it is easier for patients if the donor is anonymous rather than someone they know	4%	25%	56%	13%	2%

#### Current state of FMT research

Only 19% of respondents agreed that enough evidence for FMT exists and more than half (54%) agreed that more research is required for FMT to enter clinical practice. Less than a third (31%) consider results from using FMT in veterinary medicine to be strong evidence for performing FMT on humans. Most respondents (60%) would not consider FMT as belonging to alternative medicine and almost half (45%) considered it to be comparable with probiotics.

#### Availability and applicability

Half of the participants believed that FMT is a promising therapy for a high number of conditions. Less than a third (29%) believed that their hospital would support performing FMT.

#### Patient acceptance

Half of the respondents disagreed about feeling uncomfortable when talking to patients about FMT, however more than two thirds (69%) agreed that such discussions would be easier if FMT had a more socially acceptable name.

Almost half of the respondents (43%) believed that patients would accept FMT, however 41% were concerned about the potential of FMT to adversely impact patient dignity through social stigma and shame. More than two thirds (71%) considered that patients would be more accepting of FMT if the stool donor was anonymous and more than half (54%) believed that the existence of a stool bank would lead to increased acceptance among both patients and physicians.

#### Donor screening procedures

Almost one third (31%) of respondents considered donor screening a complex and expensive process and more than half (54%) disagreed that “the chances of someone fulfilling all the criteria of becoming a stool donor were comparable to those of being admitted to Harvard”.

#### Outcome

The concern that FMT could cause more harm than good was met with disagreement by 58% of responders and only 12% were skeptical about its long-term results.

#### Risk and side effects

Less than a quarter of respondents (17%) agreed that FMT could pose significant risk to patients, however more than two thirds (69%) believed that FMT could lead to the recipient acquiring an infection from the donor, while only 16% agreed that FMT could lead to an exacerbation of the disease being treated. As for FMT being a long and complicated process, half of the participants disagreed. Most respondents (65%) also disagreed that FMT is a riskier procedure than blood transfusion, although almost a third (29%) also disagreed that FMT is safer than standard medical treatments.

## Discussion

To our knowledge, only two studies assessing physicians’ perception of FMT have been performed [23, 24]. While its benefits have been highlighted by the recent surge in research, FMT and its applications remain topics of interest and debate, especially from methodological and ethical standpoints [25].

In our study, we found that the self-assessed degree of knowledge regarding FMT among medical students was generally low, however this may be a consequence of insufficient exposure through lectures focusing on FMT and of the lack of widespread use of the procedure. Ren et al. conducted a similar study on physicians and reported that less than half of respondents had knowledge of FMT principles and technology [24].

Respondents recognized the important role that FMT has been playing in the treatment of rCDI and IBD. Other considered indications were IBS and metabolic disorders. Our results are consistent with other published studies [24]. Improving awareness of FMT is still warranted, as 7% of respondents had no knowledge of these indications.

FMT is generally regarded as being safe, with only mild adverse effects being reported. Increased frequency of defecation, fever, abdominal pain, flatulence, vomiting and bloating were only seen during the first month and evidence suggests that their incidence is lower if the FMT preparation method used is automatic (8.7%) rather than manual (21.7%) [26]. Aside from these side effects, our respondents were also concerned about the possibility of a previously undetected infection being transmitted to the recipient, neurotransmitter imbalance or obesity. Overall, the concern that FMT could cause more harm than good was not considered to be of major significance, although one quarter of our respondents were skeptical about long-term results.

More than half of the respondents consider that FMT would be optimally administered through a lower gastrointestinal route (via colonoscope or enemas). Less than a quarter would use either oral capsules or a nasojejunal tube and the remainder had no opinion on the matter. Studies with similar results [23, 24, 27, 28] regard FMT administration via a lower gastrointestinal route not only possibly more effective due to easier colonization, but also as psychologically more acceptable.

Almost all respondents would readily recommend FMT, provided that an indication for it exists. However, only 9% would use it as a first-line treatment, while one third would only use it as a last resort and more than half would consider using it after at least one disease recurrence. More controlled clinical trials are essential to establishing clear indications and timing of administration. In our study, over half of the respondents would be willing to enroll patients in such trials, one third was undecided and the remainder would not. Several obstacles in the way of widespread use, aside from patient and physician reluctance, may be the unavailability of eligible donors (due to rigorous screening criteria) or stool banks, time-consuming preparation procedures and the possibility of FMT not being covered by insurance.

Although a specialist procedure, the shift in medical education towards active and problem-based learning [29] may benefit from an early exposure of medical students to emerging procedures such as FMT. Our results indicate that respondents are generally interested in learning more about the procedure.

A practical issue is to decide when teaching of FMT should be included in medical students’ training curriculum. As the knowledge of students on FMT is important when they start to interact with patients, we consider that this is the best moment to teach them on FMT. Depending on the structure of curricula in different medical schools, teaching on FMT should start when students begin learning gastroenterology. Teachers should be aware of the importance of offering information on new management techniques. The implementation of the teaching on FMT should be decided by chair holders of clinical diagnosis or gastroenterology. Theoretical aspects and practical demonstration on FMT would not need more than two teaching hours.

The small number of respondents, all attending a single institution, may be considered a limitation of this study. Where patients were asked for their opinion, the percentages of responders that took a neutral stance were in some cases high. This may indicate a lack of familiarity with certain aspects surrounding FMT that warrants the need for increased exposure.

## Conclusion

We found that medical students are familiar with the principles and indications of FMT and would readily recommend it. As FMT gradually enters clinical practice, we believe that more research is required to clearly identify subsets of patients that would benefit the most from FMT, as well as the optimal delivery methods and timing.

## Supplementary information

**Additional file 1.** Questionnaire regarding fecal microbiota transplantation (FMT).

**Additional file 2.** Results.

## Data Availability

All data generated or analyzed during this study are included in this published article.

## Abbreviations

CDI: *C. difficile* infection
FMT: Fecal microbiota transplantation
IBD: Inflammatory bowel disease
IBS: Irritable bowel syndrome
rCDI: Recurrent *C. difficile* infection
RCT: Randomized, controlled trial
TNFalpha: Tumor necrosis factor alpha
