# Supplementary material for: Medical students’ perception on fecal microbiota transplantation
Source: BMC Med Educ. 2019 Oct 11;19:368. doi: 10.1186/s12909-019-1804-7 (PMC6788000; doi:10.1186/s12909-019-1804-7)
Supplement: Supplementary file 1 — Additional file 1. Questionnaire regarding fecal microbiota transplantation (FMT). [file 12909_2019_1804_MOESM1_ESM.pdf]

Questionnaire regarding fecal microbiota transplantation (FMT)

* Required

1. **What is your specialty? ***

2. **How would you rate your level of knowledge regarding FMT? (indications, procedure, benefits, risks, etc.) ***

   Mark only one oval.

   - very low
   - low
   - medium
   - high
   - very high

3. **Where have you heard, seen or read about FMT? Please indicate how often you use each source. ***

   Mark only one oval per row.

	Never	Rarely	Sometimes	Frequently	Very frequently
Articles					
Courses					
Conferences					
Documentaries					
The internet					
Colleagues					
Other sources (specify below)					

4. **Other sources:**

5. **Have you indicated FMT for a patient before? ***

   Mark only one oval.

   - yes
   - no

6. **In what cases do you know of FMT to be indicated? (multiple items may be chosen) ***
Check all that apply.

- [ ] Clostridium difficile infection
- [ ] ulcerative colitis
- [ ] Crohn's disease
- [ ] irritable bowel syndrome
- [ ] obesity
- [ ] Other: ______________________

7. **What adverse effects may appear following FMT? (multiple items may be chosen) ***
Check all that apply.

- [ ] abdominal discomfort
- [ ] nausea
- [ ] cramps
- [ ] bloating
- [ ] diarrhea
- [ ] constipation
- [ ] transmission of diseases undetected by screening procedures
- [ ] neurotransmitter imbalance
- [ ] Other: ______________________

8. **Would you be willing to recomend FMT to your patients when such an indication exists? ***
Mark only one oval.

- ( ) definitely yes
- ( ) probably yes
- ( ) only after exhausting all other medical options
- ( ) probably not
- ( ) definitely not

9. **What do you consider to be the optimal timing for performing FMT? ***
Mark only one oval.

- ( ) as a first line of treatment
- ( ) after the first recurrence
- ( ) after the second recurrence
- ( ) after the third recurrence
- ( ) as a last resort
- ( ) it should never be used

10. **What do you consider to be the optimal delivery method for FMT? ***
Mark only one oval.

- colonoscopy
- enemas
- naso-jejunal tube
- frozen oral capsules
- I don't know/I don't have an opinion
- Other: ______________________

11. **Would you be willing to enroll patients in a controlled clinical study evaluating FMT efficiency? ***
Mark only one oval.

- yes
- no
- not sure

12. **Several sentences regarding FMT are presented below. Please read them carefully and then select the level that best describes your agreement with each one. ***

Mark only one oval per row.

	Strongly disagree	Somewhat disagree	Neither agree, nor disagree	Somewhat agree	Strongy agree
1. I feel uncomfortable talking to patients about FMT					
2. A more socially acceptable name would make discussions about FMT easier					
3. I believe patients would accept FMT as treatment					
4. More controlled clinical studies on FMT are needed before it can be used in clinical practice					
5. I believe there is currently enough evidence for FMT					
6. I do not believe in the long-term results of FMT					
7. FMT is a risky procedure					
8. FMT is a long and complicated process					
9. FMT can lead to transmitting of infections					
10. FMT poses a high risk of disease exacerbation					
11. FMT does not represent a permanent solution to the medical problem					
12. FMT belongs to alternative medicine					
13. I find FMT somewhat disgusting					
14. FMT is a safer treatment that standard medical treatments					
15. FMT is a riskier medical procedure than blood transfusion					
16. FMT has a negative impact on patient dignity (feelings of shame, stigma, etc.)					

	Strongly disagree	Somewhat disagree	Neither agree, nor disagree	Somewhat agree	Strongy agree
17. I believe the procedure would be supported within my institution					
18. Results from veterinary medicine represent strong evidence for using FMT in human medicine					
19. FMT can do more harm than good					
20. FMT is a therapy comparable to probiotics					
21. I believe FMT is a promising therapy for many diseases					
22. I believe this procedure would be covered by insurance in the future					
23. Donor screening is a complex and costly procedure					
24. It’s easier for someone to be “admitted to Harvard” than fulfill the criteria to become a stool donor					
25. I am interested in learning more about FMT and its practice					
26. I believe the procedure should only be carried out in medical centers specialized in FMT					
27. I believe FMT should become a usual procedure in every clinic/hospital					
28. I believe the procedure would be more accessible (duration, costs, logistics) if a stool bank existed					
29. I believe the procedure would be more easily accepted by doctors and patients if a stool bank existed					
30. I believe it is easier for patients if the donor is anonymous rather than someone they know					

13. **Would you like to add anything else regarding FMT?**
